# Continuous EEG in Critically Ill Children

**DOI:** 10.15844/pedneurbriefs-29-3-3

**Published:** 2015-03

**Authors:** Jonathan E. Kurz, Mark S. Wainwright

**Affiliations:** 1 Ruth D. & Ken M. Davee Pediatric Neurocritical Care Program, Ann & Robert H. Lurie Children's Hospital of Chicago, Chicago, IL; 2 Departments of Pediatrics and Neurology, Northwestern University Feinberg School of Medicine, Chicago, IL

**Keywords:** EEG, Critical Care, Pediatrics

## Abstract

Investigators from the Critical Care Continuous EEG Task Force of the American Clinical Neurophysiology Society reported a consensus statement on indications for the use of critical care continuous electroencephalographic monitoring (ccEEG) in adults and children.

Investigators from the Critical Care Continuous EEG Task Force of the American Clinical Neurophysiology Society reported a consensus statement on indications for the use of critical care continuous electroencephalographic monitoring (ccEEG) in adults and children [1]. The consensus statement is based on observational trials and expert opinion, and defines indications for ccEEG with the goal of early identification and treatment of neurologic pathologies that might not be apparent by clinical exam alone. This statement also provides recommendations for ccEEG duration, as well as review and interpretation frequency. ccEEG is recommended for the identification of non-convulsive seizures (NCS) and non-convulsive status epilepticus (NCSE) [2]. Specific populations at risk for NCS or NCSE include patients with persistent alteration of mental status after an acute supratentorial brain injury, after generalized convulsive status epilepticus or clinical seizures, or with encephalopathy of unknown etiology. ccEEG is also recommended in at-risk patients that require pharmacologic neuromuscular blockade, in patients with periodic discharges on a routine or emergent EEG, and for titration of continuous intravenous anticonvulsants or pharmacologically-induced coma.

Assessment of the electrographic background with ccEEG may also help predict outcome in a range of acute neurologic conditions, and could allow for early detection and treatment of cerebral ischemia in at-risk patients. ccEEG recording for at least 24 hours is recommended in most cases, although the authors acknowledge that there may be situations where shorter or longer periods of recording are necessary. Review of ccEEG by technologists is suggested as often as feasible, with interpretation by neurophysiologists at least twice daily. [1]

COMMENTARY. Observational studies in both pediatric and adult intensive care units have found frequent NCS and NCSE in critically ill patients, with rates of electrographic seizure ranging from 10 to 40% in children [3, 4]. While the use of ccEEG is increasing, ccEEG indications, duration and review frequency vary considerably between institutions [5]. In part, this reflects the limitations of the current literature; although studies suggest an association between electrographic seizure burden and poor outcomes in critically ill children [6], no randomized, controlled trials have examined the impact of ccEEG use on seizure control or patient outcomes. ccEEG can be resource-intensive, and institutions have developed varying strategies for the allocation of these resources in the absence of high-quality data to guide patient management.

This consensus statement provides a valuable starting point for development of ccEEG protocols based on the best currently available evidence. The clinical impact of ccEEG on seizure management and patient outcome remains an important area of ongoing research.
